# Guillain-Barré Syndrome Associated with SARS CoV-2 Infection: Case Report

**DOI:** 10.4314/ejhs.v32i1.21

**Published:** 2022-01

**Authors:** Dereje Nigatu, Tsion Tigabu, Meron Awraris, Andargew Yohannes, Dawit Kebede

**Affiliations:** 1 Division of Pulmonary and Critical Care Medicine, School of Medicine, Addis Ababa University, Addis Ababa, Ethiopia; 2 Eka Kotebe General Hospital, Addis Ababa Ethiopia; 3 Department of Neurology, School of Medicine, Addis Ababa University, Ethiopia

**Keywords:** Guillain Barré Syndrome, SARS CoV2, COVID 19, Neurologic symptoms

## Abstract

**Background:**

Since the outbreak of Severe Acute Respiratory Syndrome Coronavirus 2 (SARS CoV2) in December 2019, there have been some case reports of Coronavirus disease 19 (COVID 19) associated Guillain-Barré Syndrome (GBS). GBS is an inflammatory polyradiculoneuropathy associated with numerous viral and bacterial infections. Here we describe the case of an Ethiopian man with a typical clinical and electrophysiological manifestation of GBS.

**Case Presentation:**

A 70-year-old male presented with four days history of progressive and ascending bilateral limbs weakness which end up with respiratory failure. He had an antecedent headache, loss of appetite, and generalized fatigue. Electrophysiological studies showed Acute Motor and Sensory Axonal Neuropathy whereas and cerebrospinal fluid analysis revealed albuminocytologic dissociation with positive preintubation SARS CoV2 test. He was treated with supportive care and recovered successfully.

**Conclusion:**

This case illustrates one of the few occasions when patients with mild COVID-19 develop severe neurologic manifestations. Seemingly, early identification and management can improve clinical outcomes. We would like to emphasize the need to consider screening for SARS CoV-2 in patients presenting with GBS.

## Introduction

COVID 19 presents with a variety of symptoms ranging from asymptomatic to severe, rapid Multiorgan Failure (MOF), and death. Although the predominant clinical presentation is a respiratory disease, neurological manifestations are being recognized as well, and they can be central or peripheral nervous system involvements (1). Although a wide range of neurological symptoms have been described the neurological sequel of the virus remains poorly understood (1). It is also reported that COVID cases had neurological symptoms ranging from anosmia and taste disturbances to cerebrovascular accidents and seizures.

Among the rare but most devastating neurological conditions triggered by SARS-CoV-2 is GBS (2) where the evidence is limited to case reports. The new cases reported during the current pandemic have led to the recognition of GBS as a neurological complication of SARS-CoV-2, rather than being present coincidentally (3). Here, we present the case of an Ethiopian man with SARS CoV2 infection who developed GBS with respiratory failure. To our knowledge, this is the first published case of GBS associated with SARS CoV-2 infection reported from Ethiopia.

**Case presentation**: A 70 years old Ethiopian male patient with no significant prior medical history was transferred to our Hospital for ICU care after he was intubated for an indication of type II respiratory failure. His condition was preceded by headache, loss of appetite, and generalized fatigue of a week duration. Then within the subsequent 4 days, he started to experience numbness and progressive weakness involving both his upper and lower limbs, which later became completely paralyzed. He had no history of trauma, convulsions, or loss of consciousness. His physical examination was remarkable for a progressive decrease in his single breath count and quadriplegia with areflexia. During the disease course, he developed breathlessness, his single breath count dropped to less than 10, and he was intubated. The pre-intubation SARS CoV2 RT-PCR test turned out to be positive.

At the referring hospital, he was put on a mechanical ventilator (MV) with minimal to moderate support. Upon his admission to our ICU, he was responsive and alert (GCS=10T) with raised Blood Pressure (165/85). He maintained his saturation (94% with the setting: Assist Control Volume Control mode with PEEP of 8 cm of H2O, Tidal Volume of 420mL, and FiO2 of 60%). Cerebrospinal fluid (CSF) analysis result showed albuminocytologic dissociation (CSF protein 371.9mg/dl and zero cell count) with negative SARS CoV2 RT PCR from CSF. Nerve conduction test (NCT, Interpretation: All sensory responses were absent and the motor studies showed prolonged latency with drop-in Conductance velocity and amplitude including prolonged F-wave latency both in the upper and lower limb. EMG in the moving part showed reduced recruitment otherwise a normal motor unit action potential features) suggested a moderate to severe diffuse bilateral mixed (sensory>>motor) axon and demyelinating polyneuropathy suggestive of Acute Motor and Sensory Axonal Neuropathy (AMSAN) variant of GBS (Tables 1, 2 3 and 4 to show the NCT outputs); his brain and cervical Magnetic Resonance Imaging (MRIs) were normal. Chest ultrasound at admission showed normal sliding pleura/lungs with bilateral A patterns.

**Table 1:** Motor Nerve Conduction

Nerve and site	latency	Amplitude	Segment	Latency difference	Distance	Conductance velocity
**Peroneal R**						
**Ankle**	8.9ms	3.1mV	Extensor digitorum brevis- ankle	8.9ms	mm	m/s
**Fibula head**	28.6ms	0.2mV	Ankle-fibula head	19.7ms	330mm	17m/s
**Tibial L**						
**Ankle**	10.4ms	1.9	Abductor hallucis-ankle	10.4ms	mm	m/s
**Popliteal fossa**	27.3ms	0.5mV	Ankle-Popliteal fossa	16.9ms	460mm	27m/s
**Median R**						
**Wrist**	7.5	4.9	Abductor Pollicis Brevis- wrist	7.5ms	mm	m/s
**Elbow**	13.3	3.5	Wrist-Elbow	5.8ms	240mm	41m/s
**Median L**						
**Wrist**	6.0ms	2.0mV	Abductor Pollicis brevis- wrist	6.0ms	mm	m/s
**Elbow**	11.7ms	1.7mV	Wrist-Elbow	5.7ms	250mm	44m/s

**Table 2:** F-wave Studies

Nerve	M-latency	F-latency
**Peroneal R**	28.6	
**Tibial L**	27.3	77.4
**Median R**	13.3	45.9
**Median**	11.7	44.4

**Table 3:** Sensory Nerve Conduction

Nerve and site	Onset latency	Peak latency	Amplitude	Segment	Latency difference	Distance	Conductance Velocity
**Sural R**							
**Lower leg**	ms	ms	µm	Ankle-Lower leg	ms	mm	m/s
**Sural L**							
**Lower Leg**	ms	ms	µm	Ankle-lower leg	ms	mm	m/s
**Median R**							
**Wrist**	ms	ms	µm	Digit II (index finger)- wrist	ms	mm	m/s
**Ulnar R**							
**Wrist**	ms	ms	µm	Digit V(little finger)- Wrist	ms	mm	m/s
**Median L**							
**Wrist**	ms	ms	µm	Digit II(index finger)- wrist	ms	mm	m/s
**Ulnar L**							
**Wrist**	ms	ms	µm	Digit V(little finger)- wrist	ms	mm	m/s

**Table 4:** Needle EMG examination

	Insertional	Spontaneous activity			Volitional MUAPs					Max volitional activities		
**Muscle**	Insertional	Fibs	+wave	Fuse	Duration	Amplitude	Poly	Config	recruitment	pattern	amplitude	effort
**Brachioradialis** **L**	Normal	None	None	None	Normal	Normal	None	Normal	Reduced	Normal	reduced	poor
**Abductor** **Pollicis** **Brevis L**	Normal	None	None	None	Normal	Normal	None	Normal	Reduced	Normal	reduced	Sub - max

He had leukocytosis (WBC of 13,700/uL with ALC of 1,863.2/uL) and Transaminitis (ALT 3.9 times elevated and AST 3.4 times elevated); his serum electrolytes were normal and serology tests for HBV, HCV and HIV were negative. He was treated with optimal ventilatory support, dexamethasone (6mg IV daily x 10 days), prophylactic anticoagulant (Unfractionated heparin 7,500IU SQ BID), antibiotics (Cefepime), and physiotherapy.

On the sixth day of his intubation tracheostomy was done; he slowly improved (without IV immunoglobulin administration because the patient could not afford it) with his vital signs in the normal range (Blood pressure range from 115/60 to 139/81 mmHg) and after negative SARS CoV2 RT-PCR result he was discharged on the 21st hospital day with tracheostomy closed; motor strength (3/5 on LUL, 2/5 RUL, 2/5 in both lower limbs) and no longer required supplemental oxygen.

## Discussion

The clinical features of the patient with typical CSF analysis and electrophysiologic studies suggest this is GBS. The presence of SARS CoV-2 RT-PCR positivity does not appear to be a coincidence. The association of GBS with this novel virus infection has also been reported by others (1,3,5). Moreover, the patient also had an elevation of liver enzymes which are attributed to COVID 19 associated transaminitis.

Currently, cases of GBS associated with this virus have been established (2,3) and we believe that our patient too had COVID 19 associated GBS in that he had all the supporting pieces of evidence exhibited by his clinical presentation, the electrophysiological findings, and CSF analysis along with the epidemiological context and the presence of RT-PCR positivity for SARS-CoV-2 (4).

There is variability in the presentation of COVID-19 associated GBS. Some have a post-infectious (most common) or para-infectious profile, while few of them were asymptomatic for COVID-19 but developed GBS symptoms (9). Our patient developed significant neurologic manifestation a week after he developed mild COVID-19 symptoms that suggest an atypical para-infectious profile (5).

One of the early case-series studies showed neurologic manifestations in patients with COVID-19 and concluded that patients with more severe COVID-19 illness were more likely to have neurologic symptoms (1). In contrast to previous reports, our patient was relatively stable and progressively developed type II respiratory failure due to GBS, and he ended up intubated for days. This case illustrates one of the few occasions when patients with mild COVID-19 develop severe neurologic manifestations. Seemingly, serious neurologic symptoms can occur in the acute phase of COVID-19 infection with only mild symptoms. This case also emphasizes the need to consider screening for SARS CoV-2 in patients presenting with GBS in the era of this pandemic.
